# Peri-Implant Microbial Signature Shifts in Titanium, Zirconia and Ceria-Stabilized Zirconia Reinforced with Alumina Sites Subjected to Experimental Peri-Implantitis: A Preclinical Study in Dogs

**DOI:** 10.3390/antibiotics13080690

**Published:** 2024-07-24

**Authors:** Roberto López-Píriz, David Sevillano, Manuel Fernández Domínguez, Luis Alou, Natalia González, Lidia Goyos-Ball, Belén Cabal, José Serafín Moya, María Luisa Gómez-Lus, Ramón Torrecillas

**Affiliations:** 1 Advanced Oral Surgery Institute (ICOA), 28012 Madrid, Spain; lopezpiriz@gmail.com; 2 Nanomaterials and Nanotechnology Research Center (CINN-CSIC), Universidad de Oviedo (UO), Principado de Asturias, 33940 El Entrego, Spain; b.cabal@cinn.es (B.C.); jsmoya@icmm.csic.es (J.S.M.); 3 Microbiology Area-Medicine Department, School of Medicine, Universidad Complutense de Madrid, 28040 Madrid, Spain; luisalou@ucm.es (L.A.); nagonzal@ucm.es (N.G.); mlgomezl@ucm.es (M.L.G.-L.); 4 Hospitales HM, Universidad San Pablo-CEU, 28003 Madrid, Spain; mfdominguez@ceu.es; 5 Nanoker Research S.L., Polígono Industrial de Olloniego, 33660 Oviedo, Spain; l.goyos@nanoker.com (L.G.-B.); r.torrecillas@nanoker.com (R.T.)

**Keywords:** Ce-TZP/Al, zirconia, titanium, dental implants, experimental peri-implantitis

## Abstract

This study evaluates the dynamic shift in the microbiota at the peri-implant site of titanium (Ti) and zirconia (Zr) implants subjected to experimental peri-implantitis (PI) and, for the first time, of implants made of ceria-stabilized alumina-reinforced zirconia (Ce-TZP/Al), a revolutionary zirconia that is set to play a key role in modern implant dentistry. One- and two-piece (TP) implants, including Ce-TZP/AL TP/G3 glass, were placed bilaterally (six implants/side) in five beagle dogs to mimic a natural vs. ligature-induced PI following a split-mouth design. The experiment spanned 30 weeks from tooth extraction. Both PI models promoted plaque deposition at peri-implant sites. Comparatively, the PI induced by ligatures favored the deposition of anaerobes (*p* = 0.047 vs. natural). Regardless of the model, the plaque deposition pattern was entirely dependent on the implanted material. Ligated Ti and Zr implant sites accumulated up to 2.14 log CFU/mL unit anaerobic load (*p* ≤ 0.033 vs. non-ligated implant sites), predominantly comprising obligate anaerobes. Naturally occurring PI induced the deposition of co-occurring networks of obligate anaerobes and less oxygen-dependent bacteria. PI induction favored the enrichment of Ti and Zr sites with bacterial taxa belonging to the orange and red complexes (up to 28% increase naturally and up to 71% in the ligated hemiarch). Anaerobic deposition was significantly lower in ligated Ce-TZP/Al implant sites (*p* ≤ 0.014 vs. TI and Zr) and independent of the induction model (0.63–1 log units of increase). Facultative bacteria prevailed at Ce-TZP/AL sites. The abundance was lower in the Ce-TZP/AL TP implant. Unlike Ti and Zr sites, taxa from the orange and red complexes were negligible. Biofilms configured at the Ti and Zr sites after ligation-induced PI resemble those found in severe IP. We hypothesize that, although surface properties (surface energy and surface roughness) and physicochemical properties of the substrate play an important role in bacterial adhesion and subsequent plaque formation, Ce-TZP/Al modulates several biological activities that preserve the integrity of the gingival seal by limiting PI progression. In conclusion, biofilm progression differs in peri-implant sites according to the specific properties of the material. Ce-TZP/A, unlike titanium or zirconia, prevents dysbiosis in sites subjected to experimental PI and preserves the microbial signature of emergent obligate anaerobes related to PI development.

## 1. Introduction

During the last 40 years, osseointegrated implants have become a corner stone in dentistry. Currently, dental implants combine esthetics and long-term durability, but the remaining challenge lies in making progress towards the development of effective implantable materials to prevent peri-implantitis (PI), a key factor in implant failure [1,2]. The preservation of bone around implants is the main criterion in determining implant success, and peri-implant disease has become the main threat to implant health [1]. PI occurs around approximately 22–36% of all implants placed in susceptible individuals, such as smokers and subjects with poor oral hygiene, diabetes and/or a history of periodontitis [2,3].

The pathogenesis and clinical symptoms of PI resemble those of periodontitis [4]. Both diseases are chronic, inflammatory and induced by pathogenic bacterial deposition, which leads to bone resorption and, in terminal stages, to the loss of the tooth or the implant [5]. Peri-implant tissue inflammation is caused by the formation of a bacterial biofilm on the implant surface. In contrast to periodontal tissues, peri-implant tissues appear to be inadequately encapsulated to resolve progression, and plaque-associated lesions extend into marginal bone tissue, leading to subsequent progressive loss of the supporting bone [1,4,5]. Similar to periodontitis, PI is not caused by a single pathogen, but rather by the overgrowth of pathogenic bacterial taxa disrupting the balance of the biofilm microbiota at the soft tissue-implant interface [3,6]. This shift alters the symbiotic relationship between tissue and microbiota, leading to dysbiosis and triggering an excessive host-modulated inflammatory response [7,8]. The immune response and tissue destruction progression appears to be greater in peri-implant tissues as a result of a distinct microbial signature than that associated with periodontitis [3,6]. Although some known periodontitis-associated pathogens are involved in PI [3,9,10,11], peri-implant related-biofilms show more complex coagregations, with both a higher presence of bacterial taxa that are not commonly found on teeth with periodontitis and a significantly greater involvement of taxa belonging to the red and orange complexes [12]. Moreover, specific microbial signatures that depend on the severity of the PI lesions have been found to explain the distinct etiologies related to PI [6,7,13]. As PI evolves and the probing depth becomes deeper, biofilms become increasingly dysbiotic, with a lower bacterial richness and a higher prevalence of obligate Gram-negative bacterial taxa [10].

Design determines function, and the biomechanical properties of dental materials rule implant design and influence biofilm formation at the implant–soft tissue interface [1,14]. The risk of bone loss increases in two-piece dental implant systems since the microscopic gaps at the implant–abutment connection facilitate bacterial colonization [15].

Titanium and its alloys are considered the gold standard for implantology due to their high biocompatibility, favorable bone apposition/soft tissue attachment and exceptional mechanical strength [16,17]. These properties make titanium a versatile material for implant and prosthetic design [1]. However, metal corrosion is a major drawback. The release of titanium, aluminum and vanadium particles from titanium alloys leads to an undefined risk of systemic toxicity, problems in bone mineralization, allergic reactions and decreased implant strength, jeopardizing osseointegration stability [17].

As teeth are ceramic composites, shifting from metal to ceramic implants is a nature-inspired evolution, which, combined with antimicrobial and antibiotic-free coatings constitutes, an emerging strategy focused on preventing PI [18,19]. The development of zirconia (zirconium dioxide [ZrO2]) marked a significant milestone in terms of ceramic mechanical properties. Further developments based on yttria-stabilized tetragonal zirconium polycrystals (Y-TZPs) and the newer alumina-toughened zirconia (ATZ) have made ceramic implants a real alternative to titanium implants. Zirconia-based implants display high fracture strength and a reduced tendency to bacterial biofilm accumulation when compared to titanium implants [13,20]. Additionally, zirconia’s intrinsic white color fulfills the esthetic demands of patients, generally dissatisfied with metal implants in this respect [20]. Currently, Y-TZP and ATZ are the gold standard in the manufacture of ceramic oral implants, albeit the implant design constraints imposed by their low fracture toughness (K₁c value). This lack of plasticity implies that Y-TZP and ATZ implants cannot even come close to achieving the prosthetic versatility of titanium implant systems.

A new ceramic composite, consisting of ceria-stabilized zirconia reinforced with alumina (Ce-TZP/Al), shows a significantly increased fracture toughness (K₁c value) while displaying high biocompatibility and excellent osseointegration and soft tissue attachment. This outstanding mechanical behavior has come to be named “ceramic plasticity” [21]. This feature of Ce-TZP/Al enables the combination of ceramic’s biological and esthetic advantages with the high mechanical properties needed to achieve the cutting-edge designs and versatility of current metallic implants [1,16].

Biofilm removal is a prerequisite for reosseointegration. Current treatment options for removing biofilms from implant surfaces are ineffective and therefore the most effective strategy to combat PI remains the prevention of biofilm formation. Bacterial deposition on the implant is reduced by increasing adhesion resistance. Several surface properties of dental implants, such as the surface energy, roughness, topography and chemical composition of the material, have been shown to influence the attachment of microbial colonizers [1]. It has been postulated that bacterial biofilms accumulate less readily on zirconia than on titanium, which consequently reduces the risk of infection and inflammatory reactions in the adjacent soft tissue [1].

Another approach is to treat the implant surface with a coating containing cytotoxic compounds that induce the death of nearby or attached bacteria [19,22]. In this respect, coatings with bioactive glasses such as G3 (a soda–lime glass from the SiO_2_-Na_2_O-Al_2_O_3_-CaO-B_2_O_3_ system) or, to a lesser extent, ZnO_35_ (a glass belonging to the B_2_O_3_-SiO_2_-Na_2_O-ZnO system) are particularly effective in preventing the attachment of anaerobic bacteria to implant abutments and in significantly reducing bone resorption around zirconia implants [19].

Experimental models in Beagle dogs have traditionally been used to guide the therapeutic approach to peri-implantitis. This breed has a natural predisposition to accumulating oral plaque and developing gingivitis, which rapidly progresses, causing periodontal tissue breakdown [23]. Tissue destruction around implants is even more pronounced, which makes it a reference model to mimic the onset and progress of natural peri-implantitis in humans [19,22,23,24,25]. Beagle dog subgingival microbiota is closely related to that of humans [26] and their periodontal anatomy is similar, facilitating the insertion of common dental implants [19].

The submarginal insertion of ligatures around the neck of the implant promotes faster tissue breakdown (active tissue breakdown) than the natural breakdown of soft tissue by biofilm deposition after the abandonment of plaque control measures (passive tissue breakdown) [27]. Ligature-colonizing bacteria attack the junctional epithelium and peri-implant biological width, causing the deposition of a supra- and subgingival biofilm, thus triggering a process of bone destruction that, after a certain period, progresses independently of the presence of the ligature [19,23,24]. Nevertheless, the introduction of ligatures can exert a direct impact on peri-implant tissue, affecting bone resorption and potentially obscuring the actual impact of plaque accumulation on the implanted material [23,25]. Although this approach may not fully replicate the onset and progression of disease in humans, it is still regarded as an appropriate infectious model [28]. Indeed, the existing literature contains a paucity of experimental models that seek to emulate the natural progression of PI [23,29].

Experimental designs comparing ligature-induced and spontaneous PI increase our understanding of the pathology by reproducing different stages in the development of PI [29]. This inevitably leads to an increase in the number of animals used, which is contrary to the current European Union ARRIVE guidelines on the protection of animals used in scientific research, unless a split-mouth model is used to induce IP by ligation and spontaneously. A recent pilot study has highlighted a new experimental design in which ligated and non-ligated implants are placed simultaneously in the experimental animal. Despite the risk of the cross-contamination of pathogens from ligated implant sites, the model confirmed intense soft tissue inflammation, more bone resorption and higher amounts of infiltrated connective tissue and plaque accumulation in the ligated implants [28].

The aim of the current study was to comparatively evaluate the evolution of the microbial signature in the peri-implant sites of titanium and zirconia implants using an experimental model of peri-implantitis in dogs, including, for the first time, a direct comparison with implants made of Ce-TZP/Al, a material that is set to play a key role in modern implant dentistry. As a secondary objective, the effect of coating the Ce-TZP/Al substrate with G3 glass on peri-implant microbiota was evaluated. As highlighted in previous work, this antimicrobial glass prevents peri-implant disease around zirconia implants [19].

## 2. Results

### 2.1. Impact of the Peri-Implantitis Induction Model on the Pattern of Bacterial Accumulation

**Figure 1:**
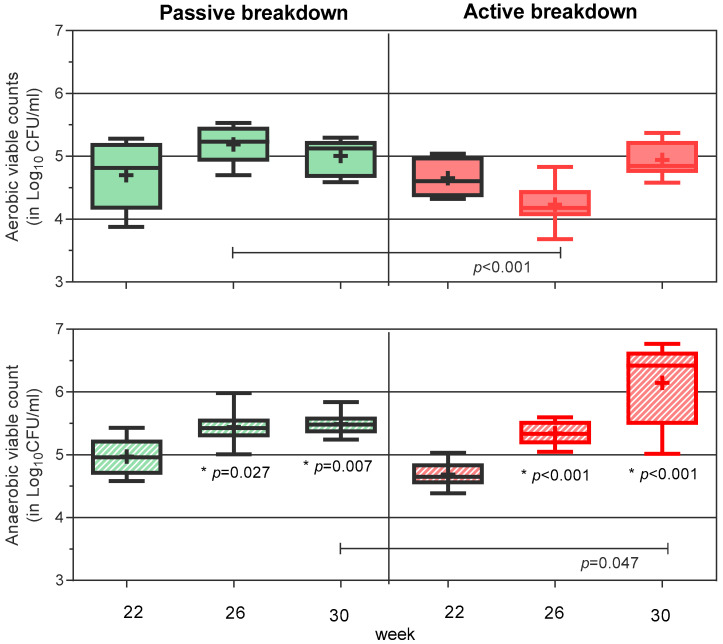
Effect of the peri-implantitis induction model on bacterial accumulation. Combined data for all implants located on the passive tissue breakdown side (green boxes) and on the active tissue breakdown side (red boxes) are shown. The red bordered box indicates samples obtained after ligature placement. Striped boxes indicate anaerobic load. Boxplot—median: line in the middle of the interquartile range, mean: +. Whiskers—minimum to maximum. * *p*-value vs. week 22.

Figure 1 illustrates that both induction models promoted bacterial growth and significant anaerobic bacterial deposition in the peri-implant sites. Aerobic and anaerobic bacterial loads per side were no different at week 22 (4.7 and 4.65 log_10_CFU/mL and 5.0 and 4.7 log_10_CFU/mL, respectively). As Figure 1 shows, the use of ligatures influenced the pattern of bacterial accumulation during the experiment, transiently decreasing the aerobic load at week 26 (*p* < 0.001) while increasing anaerobic bacteria deposition at week 30 vs. the sides undergoing passive breakdown (*p* = 0.047).

### 2.2. The Impact of Material on Peri-Implant Bacterial Accumulation

**Figure 2:**
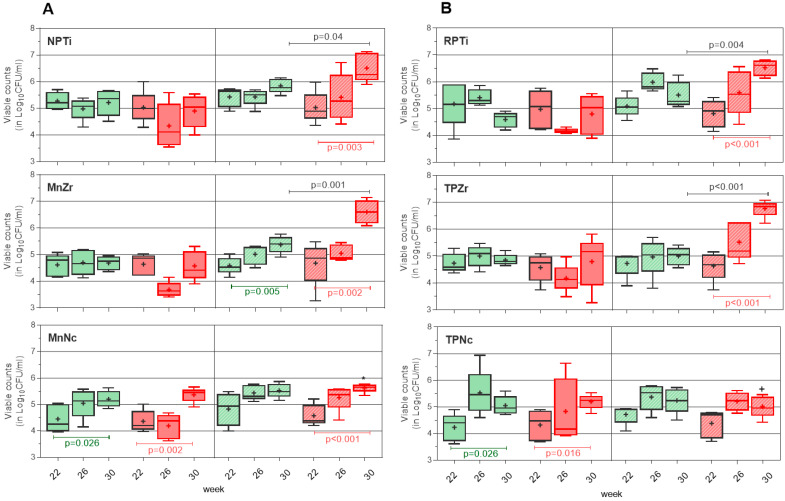
Peri-implant bacterial accumulation on different materials. (**A**). Monobloc implant. (**B**). Two-piece implants. Top box: titanium. Middle box: zirconia. Bottom box: Ce-TZP/Al. Green indicates implants on the passive tissue breakdown side and red indicates implants on the active tissue breakdown side. Filled boxes indicate aerobic load, while striped boxes indicate anaerobic load. The red-bordered box indicates samples obtained after ligature placement. Plot specifications are detailed in the caption of Figure 2. At week 22, and aside from the positioning side of the implant, the mean number of aerobically and anaerobically grown bacteria had the following ranges: NPTi, 5.0–5.3 and 5.0–5.4; RPTI, 5.0–5.2 and 4.8–5.1; MnZr, 4.4–4.5 and 4.6–4.7; TPZr, 4.6–4.7 and 4.6–4.7; MnNc, 4.4–4.5 and 4.6–4.8; and TPNc, 4.2–4.3 and 4.4–4.7. * *p* ≤ 0.01405, + *p* < 0.001 vs. titanium and zirconia implants.

Figure S1 shows indistinguishable bacterial accumulation patterns for the Y-TZP and ATZ implants. Figure 2 and Table S1 depict the peri-implant microbiota shift for the different implants, combining Y-TZP and AZT data as TPZr. As shown in Figure 2, the bacterial load was not significantly different in the peri-implant sites at week 22, although titanium accumulated a greater amount of microbiota in comparison.

After PI induction, aerobic bacteria deposition was only significant in Ce-TZP/Al sites, regardless of the PI model (*p* ≤ 0.026) and with a load increase of ~0.75–1 log units (Table S1). In the titanium sites, the aerobic load decreased by week 30.

Anaerobic bacterial population showed a gradual increase in all peri-implant sites (*p* = 0.005 for MnZr in the passive breakdown side and *p* ≤ 0.003 for titanium, zirconia and MnNc in the active breakdown side), with a significant variation in the load set at week 30 based on the PI induction model and the material used (Figure 2). On the passive breakdown side, the anaerobic load was similar on all implants at week 30, with increases of 0.3 to 0.8 log units (Table S1). In contrast, on the active breakdown side, the titanium and zirconia sites accumulated a significantly higher anaerobic load (increase of 1.5–2.14 log units, Table S1) when compared to the passive breakdown side (*p* ≤ 0.033) and in comparison to the Ce-TZP/Al implant undergoing active breakdown (*p* ≤ 0.014; 0.63–1 log units of increase, Table S1) by week 30. In the case of Ce-TZP/Al, the pattern of anaerobe accumulation was indistinguishable on both sides. Comparatively, the TPNc implant supported a lower anaerobic load by week 30, although differences were not significant vs. Ce-TZP/Al monobloc implant.

**Figure 3:**
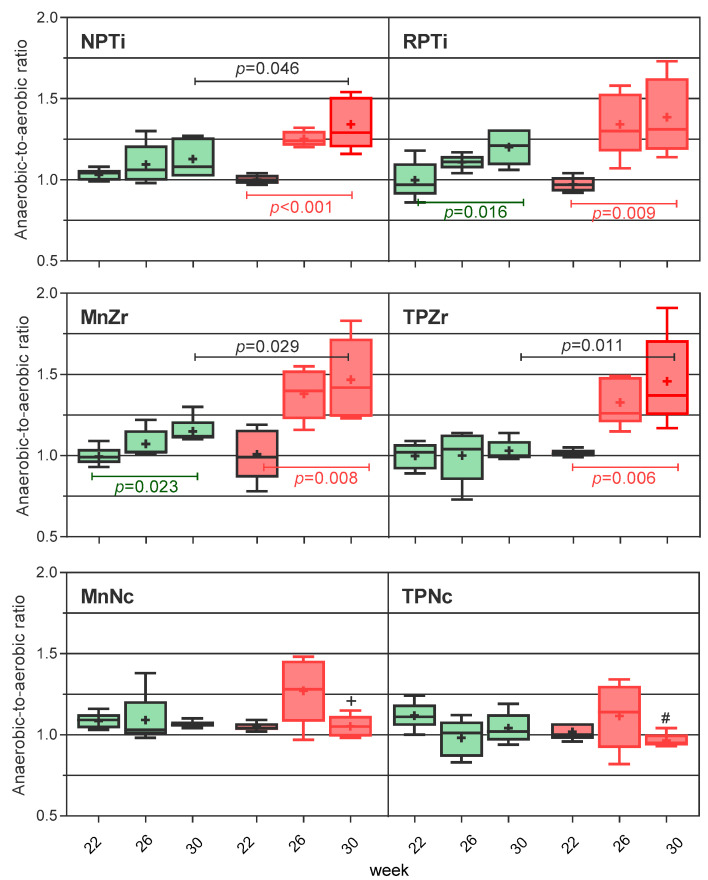
Anaerobic-to-aerobic ratio shifts at peri-implant sites. Top box: titanium. Middle box: zirconia. Bottom box: Ce-TZP/Al. Green indicates implants on the passive tissue breakdown side and red indicates implants on the active tissue breakdown side. The red-bordered box indicates samples obtained after ligature placement. Boxplot specifications are detailed in the caption of Figure 2. + *p* ≤ 0.032 vs. MnZr and TPZr at week 30. # *p* ≤ 0.046 vs. titanium and zirconia at week 30.

The anaerobic-to-aerobic (NA) ratio shifts detailed in Figure 3 indicate a progressive accumulation of obligate anaerobes in the cases of titanium and zirconia, regardless of the induction model. The higher the ratio, the greater the deposition of obligate anaerobes. By week 30, the accumulation of obligate anaerobes was notably higher on the active breakdown side (*p* ≤ 0.046 for NPTi and zirconia vs. the passive breakdown side). In Ce/TZP-Al sites, facultative anaerobe deposition prevailed regardless of the induction model, as both aerobic and anaerobic bacteria increased, while the NA ratio remained stable throughout the experiment.

### 2.3. Characterization and Dynamic Shift of Anaerobic Communities

**Figure 4:**
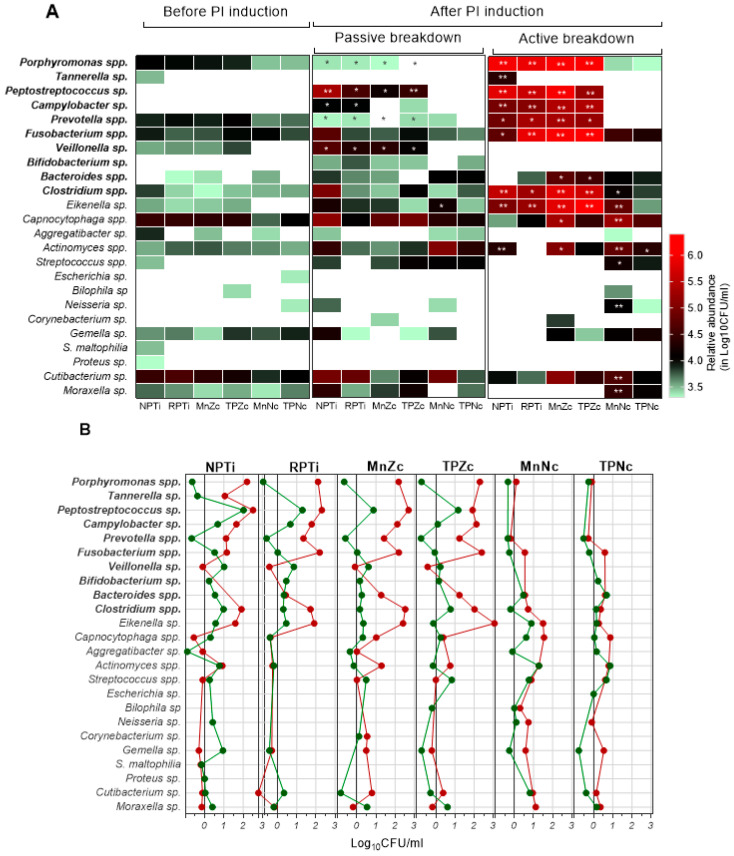
Relative abundance shifts of taxa at peri-implant sites. (**A**). Heatmap illustrating relative abundance of the taxa detected before (week 22) and after (week 30) peri-implantitis induction. No significant differences in the abundance of taxa were found on the passive tissue breakdown side and the active breakdown side at week 22. Therefore, week 22 represents the mean value of both sides. Color scale is provided in the figure. Obligate anaerobes are marked in bold. * *p* < 0.05, ** *p* < 0.01 for load at week 30 vs. week 22. (**B**). Change in the relative abundance of the taxa at peri-implant sites after passive tissue breakdown induction (green) and after active passive tissue breakdown induction (red).

Table S2 provides a comprehensive overview of the 24 anaerobic taxa identified throughout the experiment. Figure 4 and Table S3 detail the richness and relative abundance shifts of taxa at the peri-implant sites. Before PI induction, the diversity was similar in all sites despite the higher richness and abundance of anaerobic taxa found on NPTi (Table S3). Nine out of twenty taxa detected in that week—*Clostridium* spp., *Porphyromonas* spp., *Prevotella* spp., *Fusobacterium* spp. and the facultative taxa *Cutibacterium* sp., *Actinomyces* spp., *Capnocytophaga* spp., *Gemella* sp. and *Moraxella* sp.—were ubiquitously found across sites. *Eikenella* and *Veillonella* species were circumscribed for titanium and zirconia materials and *Aggregatibacter* spp. for mono-implants, while other taxa were site-specific. *Cutibacterium* sp., *Capnocytophaga* spp., *Fusobacterium* spp. and *Prevotella* spp. prevailed (~4 log_10_CFU/mL) in all sites, *Porphyromonas* spp. on titanium and zirconia, *Gemella* sp. on Ce-TZP/Al and TPZr and *Aggregatibacter* sp. around NPTi.

**Figure 5:**
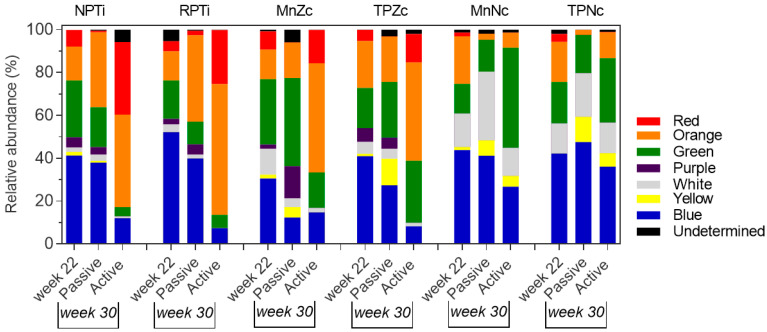
Relative abundance distribution of taxa clustered into Socransky complexes before (week 22) and after peri-implantitis induction (week 30) at peri-implant sites. Passive: passive tissue breakdown side. Active: active tissue breakdown side. Week 22 represents the mean value of both sides. The relative abundance of each complex comprised one or more bacterial taxa. Note that some taxa do not colonize the peri-implant sites of some materials (see Figure 4A). Bacterial taxa identified throughout this study and their assignments to Socransky complexes (see Table S2) are as follows: Red complex—*Porphyromonas* spp., *Tannerella* sp. Orange complex—*Peptostreptococcus* sp., *Campylobacter* sp., *Prevotella* spp., *Fusobacterium* spp. Green complex—*Eikenella* sp., *Capnocytophaga* spp., *Aggregatibacter* sp. Purple complex—*Veillonella* sp. White complex—*Actinomyces* spp. Yellow complex—*Streptococcus* spp. Blue complex—*Bifidobacterium* sp., *Bacteroides* spp., *Clostridium* spp., *Escherichia* sp., *Bilophila* sp., *Neisseria* sp., *Corynebacterium* sp., *Gemella* sp., *S. maltophilia*, *Proteus* sp., *Cutibacterium* sp., *Moraxella* sp.

After PI induction, specific groups of anaerobes were significantly enriched at the sites in a material-dependent manner. At titanium and zirconia sites, the richness of obligate anaerobes increased regardless of the PI induction model. Richness shifts for facultative bacteria were more dependent on the model, with notable decreases in the active breakdown sites. On the passive breakdown side, the obligate anaerobes *Peptostreptococcus* spp., Campylobacter spp. (titanium) or *Veillonella* sp. (titanium and MnZr) were significantly enriched (~0.5–2 log_10_CFU/mL) and abundantly found (>4 log CFU/mL) by week 30. In contrast, basal predominant taxa such as *Prevotella* spp. and *Porphyromonas* spp. or *Aggregatibacter* sp. (NPTi) were strongly displaced from the sites. Non-significant abundance shifts were observed for other taxa in comparison. As summarized in Figure 5, the taxa enriched in the titanium sites predominantly belonged to the orange complex (up to 28% increase; see supporting information in Table S4). Changes in the zirconia sites were related to multiple complexes, including the orange complex, albeit to a lesser extent than in the case of titanium (up to 5% increase; Table S4). In the active tissue breakdown sides, a lower taxa diversity (Table S3) was found for these materials. Several obligate anaerobes, including *Porphyromonas* spp., *Campylobacter* sp., *Fusobacterium* spp., *Prevotella* spp., *Peptotreptococcus* sp. and *Clostridium* spp. or *Bacteroides* spp. (zirconia) and *Tannerella* sp. (NPTi), mostly belonging to the orange and red complexes (27 to 71% increase; Table S4), as well as the facultative bacteria *Eikenella* spp. and *Capnocytophaga* spp., green complex, or *Actinomyces* spp. (NPTi, zirconia), white complex, were significantly enriched (up to 3 Log_10_CFU/mL) and found in high abundance (up to ~6 log_10_CFU/mL) by week 30. The remaining basal microbiota decreased. Some taxa that were spontaneously enriched around zirconia (*Streptococcus* sp. and *Bifidobacterium* spp.) were not observed on this side.

In Ce-TZP/Al sites, in contrast, richness shifts were related to an increase in facultative anaerobic taxa, regardless of the induction model. Diversity was higher compared with Ti and Zr sites on the active tissue breakdown side (Table S3). As shown in Figure 5 (see supporting information in Table S4), enriched taxa belonged to the green (*Eikenella* sp., *Capnocytophaga* spp.), white (*Actinomyces* spp.) or yellow (*Streptococcus* spp.) complexes. These taxa were more abundantly found around the monobloc than around the two-piece Ce-TZP/Al implant by week 30, on both the passive (increase of 0.6–1.3 vs. 0–0.9 log_10_CFU/mL) and the active tissue breakdown sides (0.9–1.5 vs. 0,3–0,9 log_10_CFU/mL). Obligate anaerobes of the orange and red complexes decreased drastically by week 30 on these implants.

## 3. Discussion

In the present study, an experimental peri-implantitis dog model with a split-mouth design was developed to assess the dynamic changes in the microbiota in titanium, zirconia and ceria-stabilized zirconia peri-implant sites subjected to both passive and active tissue breakdown. To our knowledge, this is the first experimental approach that simultaneously compares the microbiota around Ce-TZP/Al implants with those around the titanium and zirconia implants commonly used in implantology.

The insertion of ligatures around the implant neck is considered the state of the art in PI research models [19,22,23]. However, it cannot be ruled out that the mechanical trauma caused by ligatures or associated immunological reactions may overlap with the contribution of the implant surface. A natural PI induction, without the action of ligatures, appears to be more relevant to the reproduction of the onset and development of this pathology, but it also implies the need for a longer time to observe the desired results [27].

Far from achieving severe PI [27], the duration of the passive tissue breakdown in the present study was sufficient to mimic the first stages of the disease [19,23,25,27]. Previously, we demonstrated that a similar passive breakdown period increased the deposition of oral microbiota, particularly of anaerobic bacteria, in zirconia peri-implant sites, causing a bone loss of ~1.4 mm [19]. The subsequent period of active tissue breakdown (10 weeks) caused additional bone loss (221%), in accordance with a significant increase (2.36 log_10_UFC/mL) in anaerobic deposition [19].

Here, the microbiological results in zirconia sites (see Figure 1 and Table S1) were similar despite using a split-mouth design; therefore, we assume that the induced lesions should not be different. Moreover, implant ligation without a prior period of spontaneous accumulation already appears to be sufficient to cause a rapid and pronounced change in the peri-implant microbiota, significantly increasing the abundance of anaerobes vs. the sites that underwent passive breakdown (*p* ≤ 0.001) at the end of PI induction. Interestingly, in this work, we found that zirconia peri-implant sites were predominantly enriched in obligate anaerobes. Spontaneously, a limited number of obligate anaerobe taxa enriched the passive breakdown sides; however, in sides subjected to active breakdown, multiple taxa of obligate anaerobes, most of them recognized as periodontopathic pathogens belonging to the orange and red complexes [30], became the predominant inhabitants of the peri-implant region. Similar to findings in humans [31], these taxa represented a minority percentage of the initial colonizers of the peri-implant sites.

The most crucial and clinically relevant finding was that the microbial signature in titanium sites resembled that of zirconia after PI induction, whereas, in stark contrast, the Ce-TZP/Al sites evolved while accumulating mostly bacterial taxa belonging to the green, white and yellow complexes along with a decrease in the richness and abundance of obligate anaerobic taxa. The impact of ligatures on Ce-TZP/Al was virtually negligible, and the occurrence of periodontopathic bacteria taxa related to the orange or red complexes was slight.

In terms of implant configuration, two-piece implants favored the adhesion of primary colonizers [15]. Two weeks of exposure (from abutment connection) were enough to achieve a bacterial richness and abundance on this implant system equivalent to that found on monobloc implants that had been exposed to the oral microbiota for longer. The subsequent evolution of the biofilm on one- or two-piece implants was not different, regardless of the PI induction model, at least in the cases of zirconia and titanium (Figure 2 and Table S1). We assume the same progression would have occurred on both Ce-TPZ/Al implant systems if the two-piece implants had not incorporated a G3 glass coating, which had previously been shown to inhibit the growth of anaerobic bacteria on zirconia implants [19]. Our experimental design included this preventive PI strategy since we expected a different behavior on the two-piece implants. Despite the surprising results obtained on the Ce-TZP/Al monobloc implants, we found that the G3 coating improved the already favorable inhibition of anaerobic bacteria provided by this material.

The particular affinity of titanium and zirconium alloy surfaces for the oral microbiota has been well reported in vitro and in human peri-implant sites [13,18,32,33,34,35,36], but the findings for Ce-TZP/Al are entirely novel. In vitro studies have reported comparable biological properties in terms of protein adsorption, bacterial adhesion and biofilm maturation for titanium and zirconia implants, including periodontal pathogen colonization, which seems to be even superior to that of hydroxyapatite [18,33,34,35]. However, differences in the thickness and spatial organization of bacteria embedded in biofilms have been found [33]. In vivo experiments show that bacterial adhesion differs significantly [13,34,35]. A reduced bacterial adhesion on zirconia surfaces has usually been reported when submitted to spontaneous biofilm formation in healthy volunteers [13,36]. In contrast, Yamame et al. demonstrated a greater ability of titanium to inhibit the formation of aerobic biofilms, but not anaerobic ones [37]. This finding is consistent with our results, since the spontaneous biofilm accumulation revealed a greater affinity for obligate anaerobes and a certain trend to displace aerobically growing bacteria in titanium vs. zirconia peri-implant sites.

It is widely accepted, by consensus, that changes in the local microenvironment lead to shifts in microbial signatures preceding mucositis and PI [3,6,7,8,10]. Recent evidence strengthens the attribution of PI to networks of co-occurring microbes in the peri-implant site [6,10,11,12,26,38]. Furthermore, while deep PI lesions are exclusively inhabited by obligate anaerobes (*Eubacterium* spp., *Porphyromonas* spp., *Filifactor* spp., *Prevotella* spp., *Fusobacterium* spp., *Campylobacter* spp. and members of *Clostridiales* or *Peptostreptococcaceae*, among others), most of them are periodontal pathogens belonging to the red and orange complexes [10,11,12,30,38]. The early stages of peri-implant disease are associated with less dysbiotic biofilms, primarily composed of bacteria with more variable oxygen requirements (*Veillonella* spp., *Actinomyces* spp., *Cutibacterium* spp., *Streptococcus* spp., *Corynebacterium* spp., *Rothia* spp., *Neisseria* spp.) [10]. Interestingly, these latter taxa are often found in healthy volunteers in case–control studies [11].

In our view, the biofilms configured on titanium and zirconia through active tissue breakdown in the present study resemble those found in severe PI, while the networks of co-occurring obligate anaerobes with less oxygen-dependent bacteria observed spontaneously may suggest, as seen in human mucositis occurs [10], a transitional stage towards more dysbiotic biofilms. It seems clear that the insertion of ligatures favored the transition to obligate anaerobes and, therefore, the progression of PI. This explains the spontaneous displacement of certain initial colonizers, such as *Prevotella* sp. or *Phorphyromonas* sp., for example, from the zirconia and titanium peri-implant sites. These colonizers became significantly enriched on the active breakdown side. Conversely, this phenomenon clarifies the spontaneous enrichment of *Veillonella* sp. in these sites, while such enrichment was not observed on the ligature-induced peri-implantitis side.

*Veillonella* sp. has been reported to potentially contribute to titanium-related PI and has been arbitrarily detected in both PI and healthy sites [6,11,38]. This suggests that the presence of *Veillonella* sp. may serve as a biomarker of the transition toward a dysbiotic biofilm. Evaluating this statement in our study is challenging because this microorganism is less prevalent in the peri-implant site of dogs compared to humans [26,31]. In contrast, as this experiment shows, the enrichment of anaerobic taxa such as *Campylobacter* sp. and *Peptostreptococus* sp. seems to be a reliable marker of biofilm transition and, consequently, of PI progression in dog experimental models. It is important to note that microbiota research in this area and the understanding of the specific microbial dynamics and interactions involved in peri-implantitis are still evolving. However, interestingly, none of these taxa were detected on Ce-TZP/Al, suggesting that Ce-TZP/Al prevents dysbiosis in the peri-implant site. Similarly, based on the composition of transient biofilms and the lower percentage of red complex-related periodontal pathogens found after active tissue breakdown, zirconia appears to exhibit a certain protective effect compared to titanium in terms of plaque evolution [10,11,12].

It is important to note that, in the present experiment, the anaerobic-to-aerobic ratio predicted the peri-implant shift and, thus, it may be useful as a surrogate endpoint for monitoring the evolution of the peri-implant site and anticipating the onset of PI.

A correlation between implant material and the duration, in years, that implants may be in function before the development of PI has been established [7]. In agreement with other studies performed on titanium [6,39], our results suggest that the implant substrate predetermines the microbial signature in the peri-implant site and therefore the chance to progress towards PI. Corrosion debris from titanium dioxide surfaces creates a unique microenvironment capable of modifying the structure of the peri-implant microbiome [6,39]. We speculate that each material, due to its superficial and physicochemical properties, enables a particular microenvironment in which the components of the microbiota with affinity for the material will thrive. In this context, experimental PI acts by revealing this affinity. The specific microenvironment explains the divergence in biofilm evolution depending on the implanted substrate, despite the similarities in the microbiota that initially colonize the peri-implant sites.

The superficial (surface energy and surface roughness) and physicochemical properties of the substrate play an important role in bacterial adhesion and subsequent plaque formation, as occurs in cell–material integration and biocompatibility [14,34,40,41,42,43]. The neutral or positively charged surface of zirconia decreases calcium-mediated bacterial adhesion in comparison to the negatively charged titanium surfaces [34,42]. Reports concerning the surface energy of Ce-TZP/Al are scarce, but it is assumed that, as a zirconia-based material, it preserves the charge properties characteristic of zirconia.

Moderately rough surfaces are known to be the more suitable for bone osseointegration, but this feature equally promotes bacterial adhesion when compared to smoother surfaces [34,40]. The tested Ce-TZP/Al implants have an average roughness value (Ra) of 1.4 μm, which falls within the typical moderate surface roughness range of titanium implants (Ra: 1.6 μm). However, zirconia implants present a value of Ra = 0.8 μm, which is in the range of minimal surface roughness, and they are therefore less prone to bacterial and cell attachment. Nonetheless, despite the aforementioned differences in surface roughness between the implants used in this experiment, the initial bacterial load was similar in all cases. Previous studies have shown that bacterial adhesion to identically polished titanium and zirconia surfaces is the same [18], suggesting that reducing roughness on Ce-TZP/Al surfaces could potentially decrease initial bacterial attachment.

On the other hand, rough surfaces have been found to exhibit better performance in terms of osseointegration and are associated with the promotion and downregulation of leukocyte accumulation and the adhesion and secretion of proinflammatory substances [43,44]. Notably, Ce-TZP/Al implants with this surface roughness exhibited, in previous experiments, a more favorable rate of osseous integration and soft tissue attachment compared to zirconia [16,40].

The influence of the surface in experimental peri-implantitis induced by ligatures is a minor variable, since bacteria attach to and attack the peri-implant tissue from the ligature. At the molecular level, the ceria lattice generates a significant oxygen vacancy defect that facilitates the redox process and, consequently, the ability to mimic the activity of multiple enzymes involved in several biological activities, such as anti-inflammation or angiogenesis [45]. Besides osteogenic gene expression regulation, it has been reported that Ce-TZP/Al is more advantageous than titanium for promoting biological effects in the transmucosal region. Ce-TZP/Al exhibits greater human gingival fibroblast attachment, proliferation and spreading, as well as a highly upregulated expression of several extracellular matrix-related genes that might facilitate a more robust soft-tissue seal around the dental implant [46]. We concur that the capability of Ce-TZP/Al to preserve the integrity of the gingival seal is essential to limit the progression of obligate anaerobes in the peri-implant site, and, therefore, would act as a protective factor against the development of peri-implantitis in Ce- TZP/Al sites.

A major limitation of the present study was the use of culture-based microbiological techniques. Microbial richness in the peri-implant sulcus was strongly underestimated when compared to the next generation sequencing approach [6,10,13,38]. Interestingly, despite the advantages offered by these methodologies, several studies demonstrate that the detection of specific bacterial taxa does not predict peri-implantitis incidence over the years [13,36,47]. Given that PI is a dysbiotic-biofilm-related disease in which different bacterial taxa occur simultaneously, any technique that allows for the identification of changes in the peri-implant signature could be of interest to address the biological stability of different materials. On the other hand, we cannot rule out some influence of the split-mouth experimental design on the deposition of pathogenic taxa on the passive breakdown side. An increase in the oral microbiota of the relative abundance of pathogenic taxa adapted to the environment of the ligated implants was possible, conditioning the deposition of bacteria on the passive breakdown side. However, we understand this situation as a drastic lack of hygienic conditions that could equally affect different materials subjected to the action of ligatures. As our results show, the impact of this situation is minor given the significant differences in the resulting microbial signature according to the type and physicochemical characteristics of the implanted materials, irrespective of the positioning side.

In conclusion, this experiment reveals that biofilms progress differently in the peri-implant sites according to the specificity of the material that hosts the oral microbiota. The study provides a broader insight to support that new zirconia-based composites, unlike titanium or zirconia, prevent the dysbiosis of peri-implant microbiota under experimental peri-implantitis conditions. Furthermore, CeATZ/Al preserves the microbial signature of emerging obligate anaerobes associated with peri-implantitis development. These microbial behaviors around CeATZ/Al could have important implications for the long-term success of dental implants, particularly when selecting implants for patients who have a history of periodontal disease resulting in tooth or implant loss.

Ce-TZP/AL stands out as a highly promising material for dental applications, showcasing superior mechanical properties compared to conventional ceramics. It also provides the possibility to adjust its surface roughness, and, as demonstrated in this experiment, exhibits exceptional microbiological stability, which can be further enhanced through the incorporation of antibacterial glass coatings.

## 4. Materials and Methods

### 4.1. Animals

Five healthy 2-year-old male Beagle dogs were used in this study to accomplish the goal of reduction, the second of the three Rs (Replacement, Reduction and Refinement), the widely accepted ethical framework for conducting scientific experiments. The study procedures were approved by the Animal Experimentation Ethics Committee of the Jesús Usón Minimally Invasive Surgery Centre (Cáceres, Spain) (reference EXP-20190311) and were carried out in accordance with the ethical principles of the ARRIVE guidelines, the U.K. Animals (Scientific Procedures) Act 1986 and Directive 2010/63/EU for animal experiments. Animals were provided by the official suppliers of the Jesús Usón Minimally Invasive Surgery Centre. All procedures were performed under the assistance of a veterinary team and all efforts were made to minimize animal suffering. Refined dog husbandry and care was provided.

### 4.2. Experimental Procedures

This study was designed as a preclinical split-mouth randomized trial with three material implants subjected to PI by passive tissue breakdown on one mandibular hemiarch and by active tissue breakdown on the other. All procedures were performed under the effect of general anesthesia and by the same surgeon. General anesthesia was induced with 10 mg/kg intravenous propofol (Propofol Hospira, Hospira Productos Farmacéuticos y Hospitalarios, Madrid, Spain) and sustained with sevofluorano (Sevorane, Abbott Laboratories, Madrid, Spain) through a No. 7 endotracheal tube connected to a circular anesthesia circuit (Leon Plus, Heinen & Löwenstein, Bad Ems, Germany). Multimodal analgesia was employed during the perioperative period: ketorolac 1 mg/kg (Toradol 30 mg, Roche, Madrid, Spain), tramadol 1.7 mg/kg (Adolonta, Grünenthal, Madrid, Spain) and buprenorfine 0.01 mg/kg (Buprex, Reckitt, Benckiser Pharmaceuticals Limited, Berkshire, UK). The anesthetic protocol was supervised by the veterinary team.

**Figure 6:**
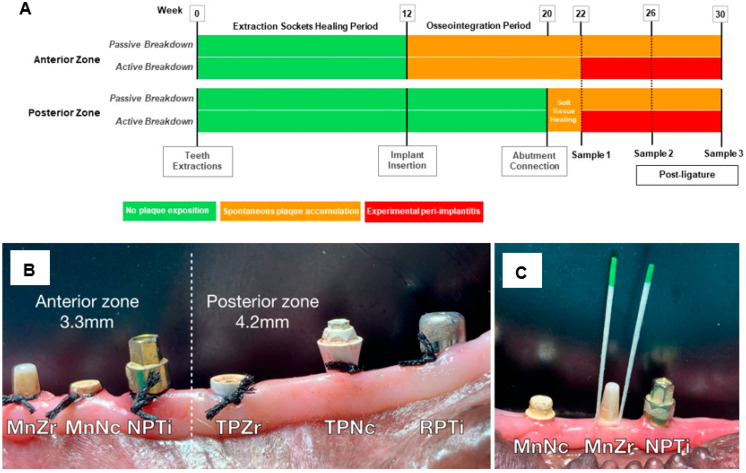
Experimental design, location of implants and sampling method. (**A**). Experiment timeline. (**B**). Implant position on anterior and posterior zone (right and left side). Anterior zone with implants of 3.3 mm in diameter and posterior zone with implants of 4.2 mm in diameter. (**C**). Subgingival sampling method. MnZr: monobloc zirconia (Strauman^®^ PURE); MnNc: monobloc Ce-TZP/Al composite (Nanoker); NPTi: narrow-plate titanium (Klockner); TPZr: two-piece zirconia (Straumann^®^ PURE, Y-TZP, or NobelPearlTM, ATZ); TPNc: two-piece Ce-TZP/Al composite (Nanoker); RPTi: regular-plate titanium (Klockner).

The timeline of the experiment is presented in Figure 6A. In all dogs, mandibular premolars and the first molar were extracted. After three months of healing, mucoperiosteal flaps were elevated and six implants were inserted, bilaterally, in each hemiarch. Monobloc (Mn) implants were placed in the most anterior zone of the edentulous regions and two-piece (TP) implants in the rearmost zone of the edentulous gap. All implants were placed according to their respective manufacturer’s instructions and with their corresponding surgical kits.

In the most anterior zone, (Figure 6B), 3 implants with a narrow diameter of 3.3 mm were inserted applying a single-stage protocol: (i) narrow plate (NP) CP titanium KL^®^ (Klockner, Barcelona, Spain), identified as NPTi, (ii) Straumann PURE^®^ ceramic monobloc (Straumann group, Basel, Switzerland), identified as MnZr and (iii) Ce-TZP/Al ceramic composite monobloc (Nanoker Research S.L., Oviedo, Spain), identified as MnNc. In the posterior zone (Figure 6B), 3 two-piece implants with a regular diameter were inserted at the alveolar bone level in a conventional two-stage surgery procedure: (i) regular plate CP titanium KL^®^ 4.2 mm. (Klockner), identified as RPTi, (ii) two-piece PURE^®^ Y-TZP 4.1 mm (Straumann) or ATZ NobelPearl^®^ 4.2 mm implants (Nobel Biocare, Kloten, Switzerland), grouped under the term TPZr and (iii) two-piece Ce-TZP/Al ceramic composite implants PR 4.2 mm. (Nanoker Research S.L.), identified as TPNc. The TPNc abutments were coated with the G3 antibacterial glassy coating [19]. Eight weeks after implant insertion surgery, transgingival and healing abutments were fitted on the TP implants and the soft tissue was left to heal for two additional weeks.

The experimental anterior zone sites were randomly allocated to the different substrates according to a computer-generated randomization list (“RANDOM()” function in Excel). This methodology ensured that the relative positioning of each material and the adjacent ones varied between experimental animals. The randomization sequence was replicated in the posterior zone and bilaterally. Allocations were concealed with sealed, numbered, tamperproof, opaque envelopes that were opened during surgery. At week 22, the active tissue breakdown period was initiated on the left side (active tissue breakdown side) following the technique described by Lindhe et al. [24]. For this purpose, cotton ligatures were placed in a sub-marginal position around the neck of the implants (Figure 6B). The ligatures were left in place until the end of experiment (week 30). The implants on the right side, without the action of ligatures, served to assess the naturally occurring microbial deposition in the peri-implant sites (passive tissue breakdown side).

From implant placement onwards, animals were fed with a soft diet. No plaque control program was implemented during the experiment. At the end of the experiments, the animals were euthanized by an overdose of potassium chloride (2 mEq/kg) under a premedication with dexme-detomidine (5 µg/kg) administered intravenously followed by an overdose of propofol (15 mg/kg) administered intravenously.

### 4.3. Collection, Sample Processing and Bacterial Quantification

Subgingival samples were obtained from the peri-implant pockets at weeks 22 (prior to ligature insertion), 26 and 30 for assessment of viable aerobic and anaerobic bacterial counts. Before sampling, the implant-surrounding tissues were dried and supragingival plaque was removed using sterile cotton pellets. Samples were collected through the insertion of two sterile paper points/site (No. 30 Maillefer, Tulsa, OK, USA), for 10 s (Figure 6C). Samples were kept at 4 °C in 1 mL of reduced transport fluid (dithiothreitol balanced mineral salt solution) until processing, which took place 6 h later in the microbiology laboratory.

A total of 180 samples were processed. Samples were vigorously vortexed (1.500 r.p.m. for 1 min), diluted (1:10–1:100,000) in phosphate-buffered saline and spiral-plated onto Columbia and Brucella agar (Becton, Dickinson and Company-BD, Franklin Lakes, NJ, USA) using an automated plating workstation (Don Whitley Scientific, Bingley, UK). Columbia agar plates were incubated at 35 °C in ambient air to quantify the aerobic bacterial load (strict aerobes and facultative anaerobes), and Brucella agar plates were incubated at 35 °C in an anaerobic chamber to isolate and quantify the anaerobic bacterial load (obligate anaerobes and facultative anaerobes). After 48 h of incubation, colony-forming units (CFU) were counted using an automated counting system (Easy Count, BioMerieux, Marcy l’Etoile, France). Results are expressed in log_10_CFU/mL. The detection limit was 1.3 log_10_ CFU/mL (20 CFU/mL).

### 4.4. Measurement of Microbiota Change at the Peri-Implant Site

The mean (±standard deviation; SD) viable bacterial count under aerobic and anaerobic conditions (in log_10_) from five animals was determined per implant at weeks 22, 26 and 30. Peri-implant bacterial load accumulation was assessed by calculating the log change in viable counts (aerobic or anaerobic) from week 22 to week 30. In addition, the anaerobic-to-aerobic bacterial ratio (N/A) [48] was determined as a surrogate endpoint of the obligate anaerobes deposition by dividing the anaerobic load by the aerobic load (in log_10_CFU/mL) at each timepoint. This ratio identifies an environment dominated by strict aerobic (ratios < 1) or obligate anaerobic bacteria (ratios > 1), since it minimizes the impact of facultative bacteria that grow simultaneously in aerobic and anaerobic conditions. Therefore, it was used to establish the dominant trend in the peri-implant environment.

### 4.5. Composition of the Anaerobic Bacterial Load

Microbiota grown under anaerobic incubation were identified and differentially enumerated combining microscopy, subculture, biochemical and molecular techniques. For this purpose, the colonies grown on Brucella agar plates from samples diluted 1:100 were used (detection limit of 2 × 10^3^ CFU/mL). Briefly, culture images were captured and digitized, into one or several fields, by using a stereo microscope (SteREO Discovery V.8, Carl Zeiss Microimaging, Oberkochen, Germany) equipped with a digital zoom camera (AxioCam ERc 5s Rev.2, Carl Zeiss Microimaging) and the processing module ZEN (Carl Zeiss Microimaging). The images were analyzed using ZEN (version 3.9) software. Colonies with different morphologies and opacity were identified and marked in the digitized fields, followed by a differential enumeration. Simultaneously, the labeled colonies were picked and subcultured on Brucella agar for an additional 48 h. Microorganisms were identified via biochemical tests (API 20 A, BioMerieux) or 16S rRNA gene sequencing. A small number of colonies that were not properly identified were included in the analysis as undetermined microorganisms. The results are expressed in CFU/mL.

The diversity estimators, Shannon’s index and richness (function Diversity in R package) and the relative abundance (in log_10_CFU/mL) of the peri-implant microbiota were recorded at each timepoint. Relative log changes were calculated from week 22 to week 30. For comparative purposes, the relative abundance distribution (in percentage) of taxa clustered into Socransky complexes [30] was expressed per implant.

### 4.6. Composition of the Anaerobic

The Shapiro–Wilk test was used to test the normality of the data. Pairwise comparison was performed using the *t*-test. The one-way ANOVA test was used to compare multiple groups. Holm–Sidak’s correction was applied to adjust the *p*-value for the multiple comparisons test. All the related statistical analyses were performed using GraphPad Prism software v 8.01 (San Diego, CA, USA).

## Data Availability

The raw data supporting the conclusions of this article will be made available by the authors on request.

## Supplementary Materials

The following supporting information can be downloaded at: https://www.mdpi.com/article/10.3390/antibiotics13080690/s1, Figure S1: Bacterial accumulation pattern of ATZ and Y-TZP implants; Table S1: Mean change in viable counts (in Log10CFU/mL ± standard deviation) at peri-implant sites by week 30; Table S2: Identification and microbiological characteristics of anaerobic bacteria isolated from peri-implant sites throughout this study; Table S3: Richness and diversity before (week 22) and after (week 30) peri-implantitis induction at peri-implant sites; Table S4: Anaerobic bacterial composition (in percentage) of peri-implant microbiota clustered into the Socransky complexes at weeks 22, 26 and 30.
