# Supplementary material for: Peri-Implant Microbial Signature Shifts in Titanium, Zirconia and Ceria-Stabilized Zirconia Reinforced with Alumina Sites Subjected to Experimental Peri-Implantitis: A Preclinical Study in Dogs
Source: Antibiotics (Basel). 2024 Jul 24;13(8):690. doi: 10.3390/antibiotics13080690 (PMC11350813; doi:10.3390/antibiotics13080690)
Supplement: Supplementary file 1 [file antibiotics-13-00690-s001.zip › antibiotics-3092412-supplementary.pdf]

## *Supplementary material*

**Figure S1. Bacterial accumulation pattern of ATZ and Y-TZP implants**

**A.** Aerobic load. **B.** Anaerobic load.

Green bars for implants located at the passive breakdown side and red bars for implant located at active breakdown side. P value for ATZ vs. Y-TZP comparison.

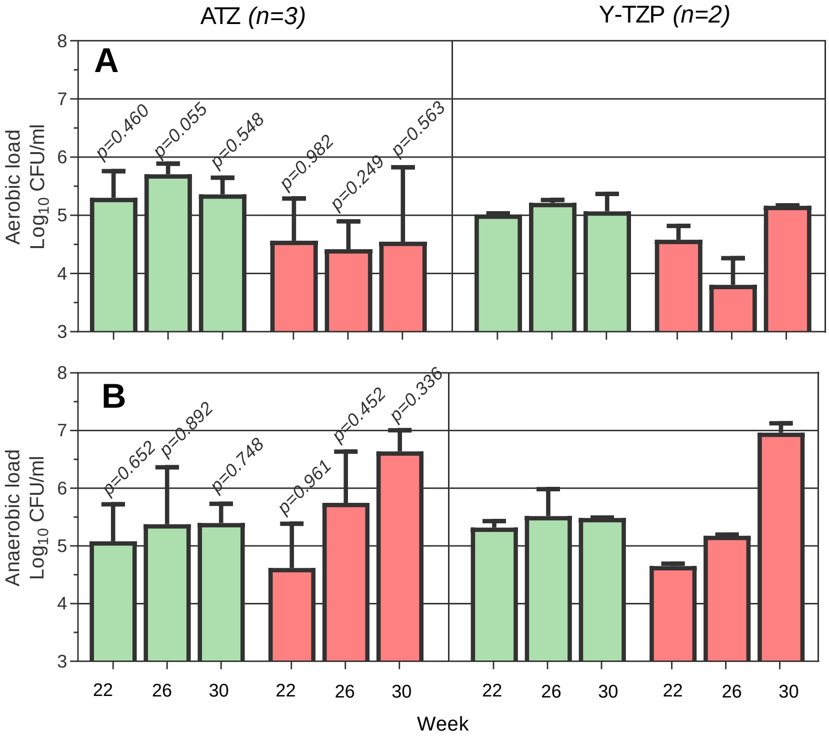


Two-piece zirconia implants are commercially available as ytria-stabilized tetragonal zirconia polycrystal (Y-TZP) or alumina-reinforced zirconia (ATZ). Y-TZP and ATZ are used interchangeably in implantology and were therefore randomly inserted in the dogs; Y-TZP in two and AZT in three dogs, both as representative materials of the two-piece zirconia implants (TPZr). As shown in Figure S1, the aerobic and anaerobic bacterial load determined in the peri-implant sulcus of both implants at weeks 22, 26 and 30 were not significantly different on the passive and active breakdown sides, indicating a similar pattern of microbiota deposition. Consequently, the data obtained for Y-TZP and ATZ were combined and referred to as TPZr implant.

**Table S1**. **Mean change in viable counts (in $Log_{10}$CFU/ml ± standard deviation) at peri-implant sites by week 30**

| Implant | *Aerobic load* | | | *Anaerobic load* | | |
|---|---|---|---|---|---|---|
| | **Passive breakdown side** | **Active breakdown side** | **[a]Log difference between quadrants** | **Passive breakdown side** | **Active breakdown side** | **[a]Log difference between quadrants** |
| **NPTi** | -0,06±0,36 | -0,13±0,69 | -0.07 | 0,32±0,45 | 1,48±0,44 | $1.16^{++}$ |
| **RPTi** | -0,59±0,58 | -0,17±0,18 | 0.41 | 0,42±0.21 | 1,71±0,62 | $1.30^{++}$ |
| **MnZr** | 0,07±0,39 | -0,06±0.77 | -0.13 | 0,79±0,54 | 1,93±0,83 | $1.14^{+}$ |
| **TPZr** | 0,12±0,53 | 0,22±0,59 | 0.07 | 0,27±0,70 | 2,14±0,34 | $1.87^{++}$ |
| **MnNc** | 0,75±0,65 | 1,00±0,38 | 0.26 | 0,70±0,65 | 1,05±0,36 | 0.35 |
| **TPNc** | 0,81±0,55 | 0,88±0,61 | 0.00 | 0,52±0,58 | 0,63±0,48 | 0.10 |

Negative values indicate a decrease in the bacterial load at week 30 vs. week 22

[a]Viable count in the active breakdown site – passive breakdown side (in $log_{10}$ CFU/ml)

$^{+}p<0.05$, $^{++}p<0.01$

**Table S2. Identification and microbiological characteristics of anaerobic bacteria isolated from peri-implant sites throughout the study.**

| *Microorganisms* | *Obligate anaerobe[#]* | *Gram stain and Morphology* | *Assignment to Socransky microbial complex* |
|---|---|---|---|
| *Porphyromonas* spp.* | yes | Gram negative rod | *Red* |
| *Tannerella forsithia* (*Tannerella* sp.) | yes | Gram negative rod | *Red* |
| *Peptostreptococcus* sp. | yes | Gram positive cocci | *Orange* |
| *Campylobacter rectus* (*Campylobacter* sp.) | yes | Gram negative rod | *Orange* |
| *Prevotella* spp.* | yes | Gram negative rod | *Orange* |
| *Fusobacterium* spp.* | yes | Gram negative rod | *Orange* |
| *Veillonella parvula* (*Veillonella* sp.) | yes | Gram negative cocci | Purple |
| *Bifidobacterium* sp. | yes | Gram negative rod | Blue |
| *Bacteroides* spp.* | yes | Gram negative rod | Blue |
| *Clostridium* spp.* | yes | Gram positive rod | Blue |
| *Eikenella corrodens* (*Eikenella* sp.) | no | Gram negative rod | Green |
| *Capnocytophaga* spp.* | no | Gram negative rod | Green |
| *Aggregatibacter actinomycetemcomitans* (*Aggregatibacter* sp.) | no | Gram negative rod | Green |
| *Actinomyces* spp.* | no | Gram positive rod | White |
| *Streptococcus* spp.* | no | Gram positive cocci | Yellow |
| *E. coli* (*Escherichia* sp.) | no | Gram negative rod | Blue |
| *Bilophila wadsworthia* (*Bilophila* sp.) | no | Gram negative rod | Blue |
| *Neisseria* sp. | no | Gram negative rod | Blue |
| *Corynebacterium* sp. | no | Gram positive rod | Blue |
| *Gemella morbillorum* (*Gemella* sp.) | no | Gram positive cocci | Blue |
| *S. maltophilia* | no | Gram negative rod | Blue |
| *Proteus* sp. | no | Gram negative rod | Blue |
| *Cutibacterium* sp. | no | Gram positive rod | Blue |
| *Moraxella canis* (*Moraxella* sp.) | no | Gram negative rod | Blue |

*2-3 species belonging to the genus were isolated and identified

[#]based on isolated genus member

Throughout the experimental period, 40 bacterial species belonging to 24 different genera were identified at densities ≥ 2x103 CFU/ml (limit of quantification of the assay). This limit of quantification ensured species isolation and differential counting of bacteria on Brucella agar plates.

The predominant group included several anaerobic facultative species belonging to 14 different genera; 9 of Gram-negative rods associated with the green (*Eikenella* sp, *Capnocytophaga* spp. and *Aggregatibacter* sp.) and blue microbial complexes proposed by Socransky (Socransky et al, 1998), 3 genera of Gram-positive rods associated with the white (*Actinomyces* spp.) and blue (*Cutibacterium* sp. and *Corynebacterium* sp.) complexes, and 2 genera of Gram-positive cocci associated with the yellow (*Streptoccocus* sp.) and blue (*Gemella* sp.) complexes.

The obligate anaerobic species included 10 different genera, mainly Gram negative rods associated with the red and orange microbial complexes (*Porphyromonas* spp., *Tannerella* sp., *Campylobacter* sp., *Prevotella* spp., *Fusobacterium* spp.). The remaining obligate anaerobic species were: gram-positive cocci (*Peptostreptococcus* sp.) belonging to a genus aligned with the orange complex, gram-negative cocci (*Veillonella parvula*) aligned with the purple complex, and gram-negative (*Bifidobacterium* sp. and *Bacteroides* sp.) and gram-positive (*Clostridium* sp.) rods aligned with the blue complex.

The correspondence between the detected taxa and the Socransky complexes is indicated in the right column. The unrelated bacteria were included in the blue complex.

**Table S3. Richness and diversity before (week 22) and after (week 30) peri-implantitis induction at peri-implant sites**

| Implant | *Before PI induction** | | | | *After PI induction* | | | | | | | |
|---|---|---|---|---|---|---|---|---|---|---|---|---|
| | | | | | **Passive Breakdown side** | | | | **Active Breakdown side** | | | |
| | **Richnnes** | | | *Shannon-Wiener Index* | **Richnnes** | | | *Shannon-Wiener Index* | **Richnnes** | | | *Shannon-Wiener Index* |
| | ***Anaerobes*** | ***Facultative*** | ***Obligate anaerobes*** | | ***Anaerobes*** | ***Facultative*** | ***Obligate anaerobes*** | | ***Anaerobes*** | ***Facultative*** | ***Obligate anaerobes*** | |
| **NPTi** | 16 | 10 | 6 | 2,357 | 18 | 9 | 9 | 2,22 | 11 | 4 | 7 | 1,590 |
| **RPT** | 12 | 6 | 6 | 1,962 | 15 | 6 | 9 | 2,088 | 10 | 3 | 7 | 1,576 |
| **MZr** | 12 | 7 | 5 | 1,954 | 14 | 7 | 7 | 1,981 | 13 | 6 | 7 | 1,843 |
| **TZr** | 12 | 8 | 5 | 2,186 | 14 | 7 | 7 | 2,041 | 12 | 5 | 7 | 1,434 |
| **MNc** | 11 | 6 | 5 | 2,308 | 12 | 8 | 4 | 2 | 14 | 10 | 4 | 2,044 |
| **TNc** | 11 | 7 | 4 | 2,269 | 12 | 8 | 4 | 1,971 | 12 | 8 | 4 | 2,14 |

*Taxa were detected in both sides. No significantly differences in abundance were found

**Table S4. Anaerobic bacterial composition (in percentage) of peri-implant microbiota clustered into the Socransky complexes at weeks 22, 26 and 30.**

Positive values (in bold) in the change column indicate relative increase of the complex in the population.

| Implant | Complex | Passive Breakdown Side | | | | Active Breakdown Side | | | |
|---|---|---|---|---|---|---|---|---|---|
| | | *Week 22* | *Week 26* | *Week 30* | *Change at week 30 vs. week 22* | *Week 22* | *Week 26* | *Week 30* | *Change at week 30 vs. week 22* |
| **NPTi** | Blue | 41.98 | 27.70 | 37.99 | *-3.99* | 40.56 | 22.86 | 12.06 | *-33.43* |
| | Yellow | 1.34 | 0.46 | 0.77 | *-0.57* | 1.86 | 0.00 | 0.00 | *-2.06* |
| | White | 2.90 | 8.14 | 2.92 | ***0.02*** | 1.59 | 4.26 | 0.80 | *-1.05* |
| | Purple | 4.36 | 2.61 | 3.58 | *-0.78* | 5.12 | 0.00 | 0.00 | *-5.85* |
| | Green | 28.35 | 10.92 | 18.52 | *-9.83* | 24.64 | 3.44 | 4.37 | *-23.59* |
| | Orange | 15.55 | 43.09 | 35.02 | ***19.47*** | 16.09 | 43.74 | 43.19 | ***32.56*** |
| | Red | 5.51 | 7.09 | 0.72 | *-4.79* | 9.91 | 22.20 | 33.80 | ***27.91*** |
| | ND | 0.00 | 0.00 | 0.49 | | 0.23 | 3.50 | 5.78 | |
| **RPTi** | Blue | 52.47 | 35.16 | 39.89 | *-12.58* | 51.88 | 8.98 | 7.40 | *-48.15* |
| | Yellow | 0.00 | 0.22 | 0.00 | *0.00* | 0.00 | 0.00 | 0.00 | *0.00* |
| | White | 4.72 | 5.05 | 1.83 | *-2.89* | 2.77 | 0.00 | 0.00 | *-2.86* |
| | Purple | 1.08 | 1.09 | 4.80 | ***3.72*** | 3.83 | 0.00 | 0.00 | *-3.95* |
| | Green | 16.92 | 3.33 | 10.60 | *-6.32* | 18.79 | 8.11 | 6.14 | *-13.37* |
| | Orange | 12.13 | 54.63 | 40.25 | ***28.12*** | 15.02 | 60.69 | 61.03 | ***49.12*** |
| | Red | 4.87 | 0.52 | 2.09 | *-2.78* | 4.62 | 22.15 | 25.15 | ***22.18*** |
| | ND | 7.80 | 0.00 | 0.54 | | 3.09 | 0.07 | 0.27 | |
| **MnZr** | Blue | 27.28 | 39.84 | 12.39 | *-14.89* | 39.36 | 7.86 | 14.75 | *-19.15* |
| | Yellow | 1.79 | 3.37 | 4.81 | ***3.02*** | 2.10 | 0.00 | 0.00 | *-1.77* |
| | White | 15.00 | 1.95 | 4.09 | *-10.91* | 10.56 | 1.76 | 2.15 | *-7.11* |
| | Purple | 2.35 | 4.02 | 15.08 | ***12.73*** | 1.85 | 0.00 | 0.00 | *-1.59* |
| | Green | 33.09 | 27.97 | 41.11 | ***8.02*** | 32.41 | 14.66 | 16.50 | *-11.30* |
| | Orange | 11.30 | 22.68 | 16.47 | ***5.17*** | 10.87 | 57.61 | 50.95 | ***34.65*** |
| | Red | 9.19 | 0.00 | 0.00 | *-9.19* | 0.93 | 14.37 | 15.52 | ***7.76*** |
| | ND | 0.00 | 0.17 | 6.05 | | 1.91 | 3.73 | 0.13 | |
| **TPZr** | Blue | 42.54 | 32.05 | 27.44 | *-15.10* | 46.35 | 12.70 | 8.18 | *-31.08* |
| | Yellow | 0.00 | 0.00 | 12.30 | *12.30* | 2.98 | 0.00 | 0.00 | *-2.41* |
| | White | 8.75 | 0.69 | 4.66 | *-4.09* | 2.98 | 0.00 | 1.70 | *-0.71* |
| | Purple | 5.60 | 3.68 | 5.31 | *-0.29* | 8.73 | 0.00 | 0.00 | *-7.29* |
| | Green | 21.00 | 28.03 | 25.86 | ***4.86*** | 19.44 | 16.69 | 28.97 | ***12.64*** |
| | Orange | 17.95 | 31.53 | 21.20 | ***3.25*** | 17.22 | 49.27 | 45.87 | ***20.11*** |
| | Red | 4.15 | 2.02 | 0.00 | *-4.15* | 2.21 | 21.05 | 13.22 | ***6.76*** |
| | ND | 0.01 | 2.00 | 3.24 | | 0.10 | 0.29 | 2.07 | |
| **MnNc** | Blue | 48.77 | 54.92 | 41.14 | *-7.63* | 38.82 | 22.65 | 26.77 | *-12.05* |
| | Yellow | 0.00 | 0.00 | 7.15 | ***7.15*** | 3.03 | 0.00 | 4.95 | ***1.92*** |
| | White | 15.91 | 3.55 | 32.14 | ***16.23*** | 15.33 | 9.97 | 13.11 | *-2.23* |
| | Purple | 0.00 | 0.00 | 0.0 | *0.0* | 0.00 | 0.00 | 0.00 | *0.00* |
| | Green | 10.37 | 34.17 | 14.77 | ***4.40*** | 17.19 | 29.07 | 46.74 | ***29.55*** |
| | Orange | 22.80 | 5.94 | 2.90 | *-19.90* | 21.65 | 34.36 | 6.97 | *-14.68* |
| | Red | 2.15 | 1.42 | 0.00 | *-2.15* | 1.19 | 3.95 | 0.22 | *-0.97* |
| | ND | 0.00 | 0.00 | 0.81 | | 2.78 | 0.00 | 1.23 | |
| **TPNc** | Blue | 47.49 | 60.44 | 47.52 | ***0.04*** | 36.87 | 24.69 | 36.11 | *-0.75* |
| | Yellow | 0.00 | 0.00 | 11.76 | ***11.76*** | 0.42 | 0.00 | 6.22 | ***5.80*** |
| | White | 5.75 | 6.22 | 20.43 | ***14.68*** | 21.88 | 7.34 | 14.35 | *-7.53* |
| | Purple | 0.00 | 0.00 | 0.00 | *0.00* | 0.00 | 0.00 | 0.00 | *0.00* |
| | Green | 19.00 | 20.79 | 17.95 | *-1.05* | 19.73 | 26.83 | 29.98 | ***10.25*** |
| | Orange | 23.31 | 10.29 | 2.25 | *-21.06* | 14.16 | 35.97 | 12.12 | *-2.04* |
| | Red | 4.45 | 2.25 | 0.00 | *-4.45* | 2.97 | 5.18 | 0.23 | *-2.74* |
| | ND | 0.00 | 0.00 | 0.08 | | 3.97 | 0.00 | 0.99 | |
